# Effective targeting of breast cancer cells (MCF7) via novel biogenic synthesis of gold nanoparticles using cancer-derived metabolites

**DOI:** 10.1371/journal.pone.0240156

**Published:** 2020-10-06

**Authors:** Sameh S. M. Soliman, Tasneem B. Alhamidi, Shifaa Abdin, Ahmed M. Almehdi, Mohammad H. Semreen, Razan B. Alhumaidi, Sarra B. Shakartalla, Mohamed Haider, Mohamed I. Husseiny, Hany A. Omar

**Affiliations:** 1 Research Institute for Medical and Health Sciences, University of Sharjah, Sharjah, UAE; 2 Department of Medicinal Chemistry, College of Pharmacy, University of Sharjah, Sharjah, UAE; 3 Faculty of Pharmacy, Zagazig University, Zagazig, Egypt; 4 Department of Chemistry, College of Sciences, University of Sharjah, Sharjah, UAE; 5 Department of Pharmaceutics and Pharmaceutical Technology, College of Sciences, University of Sharjah, Sharjah, UAE; 6 Department of Pharmaceutics and Industrial Pharmacy, Faculty of Pharmacy, Cairo University, Cairo, Egypt; 7 Department of Translational Research & Cellular Therapeutics, Beckman Research Institute of City of Hope, Duarte, CA, United States of America; 8 Department of Pharmacy Practice and Pharmacotherapeutics, College of Sciences, University of Sharjah, Sharjah, UAE; 9 Department of Pharmacology, Faculty of Pharmacy, Beni-Suef University, Beni Suef, Egypt; VIT University, INDIA

## Abstract

Biogenic synthesis of nanoparticles provides many advantages over synthetic nanoparticles including clean and non-toxic approaches. Nanoparticle-based application for the development of diagnostics and therapeutics is a promising field that requires further enrichment and investigation. The use of biological systems for the generation of gold nanoparticles (AuNPs) has been extensively studied. The search for a biocompatibility approach for the development of nanoparticles is of great interest since it can provide more targeting and less toxicity. Here, we reported a bio-reductive approach of gold to AuNPs using metabolites extracted from mammalian cells, which provided a simple and efficient way for the synthesis of nanomaterials. AuNPs were more efficiently synthesized by the metabolites extracted from breast cancer (MCF7) and normal fibroblasts (F180) cells when compared to metabolites extracted from cell-free supernatants. The metabolites involved in biogenic synthesis are mainly alcohols and acids. Spectroscopic characterization using UV-visible spectra, morphological characterization using electron microscopy and structural characterization using X-ray diffraction (XRD) confirmed the AuNPs synthesis from mammalian cells metabolites. AuNPs generated from MCF7 cells metabolites showed significant anticancer activities against MCF7 and low toxicity when compared to those generated from F180 cells metabolites. The results reflected the cytotoxic activities of the parent metabolites extracted from MCF7 versus those extracted from F180. Comparative metabolomics analysis indicated that MCF7-generated AuNPs harbored tetratetracontane, octacosane, and cyclotetradecane while those generated from F180 harbored a high percentage of stearic, palmitic, heptadecanoic acid. We related the variation in cytotoxic activities between cell types to the differences in AuNPs-harboring metabolites. The process used in this study to develop the nanoparticles is novel and should have useful future anticancer applications mainly because of proper specific targeting to cancer cells.

## Introduction

Nanotechnology gains lots of interest particularly those employing the use of AuNPs in medical applications. AuNPs showed remarkable potential in diagnostic and therapeutic purposes, including biosensor applications, targeted delivery of anticancer drugs, bio-imaging of cells and tissues, and immunoassays [1]. AuNPs showed superior preference in medical applications when compared to other metal nanoparticles, particularly because of low toxicity [2].

Reduction of Au^3+^ ions to AuNPs by biological systems offers clean, nontoxic and eco-friendly synthetic technology [3]. Control synthesis of biocompatible metal nanoparticles using yeast, fungi, bacteria and plants [4, 5], encourage the use of mammalian cells to develop nanoparticles. It has been reported that AuNPs can be generated in situ after incubation of Au^3+^ with mammalian cell cultures [6]. Furthermore, it has been observed that nanoparticles can be generated inside the epithelial cells and in intact tumor tissues [6].

The potential toxic impact of AuNPs is controversial, although recent data indicated that cytotoxicity of AuNPs depends on the type of the cell [7] as well as the size and level of aggregation of those nanoparticles [8]. In order to avoid toxicity of NPs and for proper targeting, scientists have employed the use of biological ligands such as proteins, polysaccharides, aptamers, peptides, and small molecules to develop NPs with specific capping [9]. However the use of biological ligands from cancer cells to target its own has never been studied. Therefore, we assumed that generation of AuNPs using mammalian cell metabolites may offer new insight in cancer therapy, since metabolites are the end products of all biological processes of a cell. Additionally, using cancer versus normal cell metabolites may afford special aggregation of the AuNPs with unique capping metabolites that allow variable cytotoxic activities between cell types. The use of cancer cell metabolites to develop NPs as anticancer agents for possible targeting is novel and has never been employed.

## Materials and methods

### Mammalian cell lines

The cells used in this study included breast cancer cells (MCF7, American Type Culture Collection, Virginia, USA) and normal fibroblast cells (F180, kindly provided by Dr. Ekkehard Dikomey, University Cancer Center, Hamburg University, Hamburg, Germany). The cells were maintained in Dulbecco's Modified Eagle Medium (DMEM) media supplemented with 10% fetal bovine serum (FBS) at 37°C in a humidified atmosphere containing 5% CO_2_ under effective aseptic techniques to prevent any possible contamination and according to [10]. The cells were checked every day for confluency and mycoplasma contamination. The positive cultures were immediately discarded and followed by full decontamination of the laboratory.

### Mammalian cells preparation and metabolites extraction

Mammalian cell cultures were maintained in DMEM media supplemented with 10% FBS and incubated at 37°C in a humidified atmosphere containing 5% CO_2_ in T-75 Corning cell culture flasks with vent cap (Sigma) until confluency. The cultures were then centrifuged at 19000 RCF for 15 min in order to separate the cultures into cells and cell-free supernatants fractions. The supernatants were collected and extracted by 100% ethyl acetate (2 times), while the cells (~4x 10^6^) were ground with a pestle and extracted with 100% methanol (1 time). Both extracts were filtered using filter paper and the organic solvents were separated and evaporated using a rotatory evaporator at 45 ^o^C [11]. On the other hand, the metabolites capped the formed gold nanoparticles were extracted by sonicating the NPs with methanol for 15 min at room temperature. The solutions were filtered and the methanol was evaporated. The obtained metabolites residues were either analysed by GC-MS or tested on mammalian cells. All metabolite extracts were described in Table 1.

**Table 1:** Descriptions of the metabolites extracts employed in the study.

Extract symbol	Description
**SE1**	Ethyl acetate extract of the supernatant collected from fibroblasts cell cultures
**SE2**	Ethyl acetate extract of the supernatant collected from MCF7 cell cultures
**CE1**	Methanol extract of fibroblasts cells
**CE2**	Methanol extract of MCF7 cells
**SNE1**	Ethyl acetate extract of the supernatant collected from fibroblasts cell-generated AuNPs
**SNE2**	Ethyl acetate extract of the supernatant collected from MCF7 cell-generated AuNPs
**NE1**	Methanol extract of fibroblast cells-generated AuNPs
**NE2**	Methanol extract of MCF7 cells-generated AuNPs

### Synthesis of AuNPs

Gold chloride solution (1mM) was prepared in phosphate-buffered saline (PBS). The metabolite extract (400μg) was suspended in 1 mL PBS solution, filtered through 0.2μm filters and mixed with 100 μL gold chloride solution. The mixture was incubated in 12-well corning plate for 6 days in 37°C. Control experiments were maintained using gold chloride solution in PBS or metabolites extract in PBS without gold chloride. The endpoint was identified when the solution turned in color most likely to purple [12]. In order to collect the nanoparticles, the plates were shaken for 5 min and then decanted in Eppendorf tubes. The tubes were centrifuged at 19000 RCF for 30 min and the supernatant was decanted to a new Eppendorf tube. The collected nanoparticles were further washed with PBS, followed by centrifugation and the nanoparticles were collected and named “AuNPs”. The gold nanoparticles (AuNPs) were stored at concentration 50 μg/ mL in fresh PBS at 4°C for a period of 4 months.

### Characterization of AuNPs

The synthesized AuNPs were identified using UV/visible spectroscopy by measuring the absorbance of gold at 200–900-nm wavelength range using UV-Vis spectrophotometer (Spectro UV-2510TS; Labomed, Inc.). The shape and surface topology of AuNPs were examined by scanning electron microscope (SEM) using TESCAN VEGA4 XM SEM (SE Detector, 30 kV, high vacuum) and transmission electron microscopy (TEM) using JEOL-@2100, JeolLtd, Japan. The samples were placed on standard carbon-coated copper grids (200-mesh) and air-dried for about 2h prior to TEM measurement. The energy-dispersive X-ray spectroscopy (Oxford Instruments X-Max 50 EDS detector) was carried out according to Al-Nuairi et al. [13]. X-ray diffraction (XRD) measurements were carried out using Cu radiation on a Bruker D8 Advance Powder Diffractometer.

The average particle size (z-ave) and size distribution of the synthesized AuNPs were determined by photon correlation spectroscopy at a scattering angle of 90°. Surface charge was measured following principles of laser Doppler velocimetry and phase analysis light scattering (M3-PALStechnique). Measurements were carried out on samples after 10x dilution with milliQ deionized water at room temperature using Zetasizer Nano ZS (Malvern Instrument, UK) and according to our protocol [14].

### Cell cytotoxicity using MTT staining

The cell cytotoxicity was performed using 3-(4, 5-dimethyl thiazolyl-2)-2,5-diphenyltetrazolium bromide (MTT) assay as described in [15]. Briefly, 96-well micro-plates were seeded with 4000 cells/ well of either breast cancer (MCF7) or normal fibroblast cells (F180) and incubated for 24 h at 37°C in a humidified atmosphere containing 5% CO_2_. In a pilot experiment, metabolite extracts and AuNPs at concentrations 1, 10, 50, 100 μg/ mL were suspended in mammalian cells media and filter sterilized using 0.22 filters prior to application on seeded cells. Only concentration 10 μg/ mL was selected for further investigation based on the significant difference in activity obtained and according to previously published data [16]. The cultures were incubated for 16 h. A 200μL MTT (0.5mg/ mL) reagent was added to each well. The plates were incubated 2–4 h and the liquid was then discarded followed by addition of 200μL DMSO to each well. The endpoint of the experiment was indicated by the appearance of purple color. The color was measured using Multiskan Go machine (Spectrophotometer) at 570nm. Each experiment was done in duplicate, each with 6 repeats. Cell viability was calculated using the following formula adapted from [17] and according to our publication [14].

%oflivingcells=(ODexperimental)/(ODcontrol)×100

### Gas Chromatography-Mass Spectrometry (GC-MS)

GC-MS analysis was performed as described before [18]. The metabolite extracts were derivatized by adding 50 μL of *N*-trimethylsilyl-*N*-methyl trifluoroacetamide and trimethylchlorosilane (MSTFA + 1% TMS) followed by incubation at 50°C for 30 min prior to GC-MS analysis. The derivatized samples were injected into QP2010 gas chromatography-mass spectrometer (GC-2010 coupled with a GC-MS QP-2010 Ultra) equipped with an auto-sampler (AOC-20i+s) from Shimadzu (Tokyo, Japan), using Rtx-5ms column (30 m length × 0.25 mm inner diameter × 0.25 μm film thickness; Restek, Bellefonte, PA). Data collection and analysis were performed using MSD Enhanced Chemstation software (Shimadzu). Product spectra were identified by comparison of the measured fragmentation patterns to those found in the NIST 08 Mass Spectral Library.

### Statistical analysis

The data was collected and graphed using Graph Pad Prism (5.04, La Jolla, CA, USA). The effects of different extracts and AuNPs on cancer and fibroblast cell lines were analyzed by two-way analysis of variance (ANOVA). The statistical significance was calculated with Bonferroni’s multiple comparisons test and significance level indicated by asterisks (*, *P*<0.05; **, *P*<0.01: ***, *P*<0.001; ****, *P*<0.0001). The data display the mean of the percentage of the survival rate of mammalian cells ± SEM of 6 replicas.

## Results

### Metabolites extracted from MCF7 cancer cells showed potential cytotoxic activities on their own

**Fig 1:**
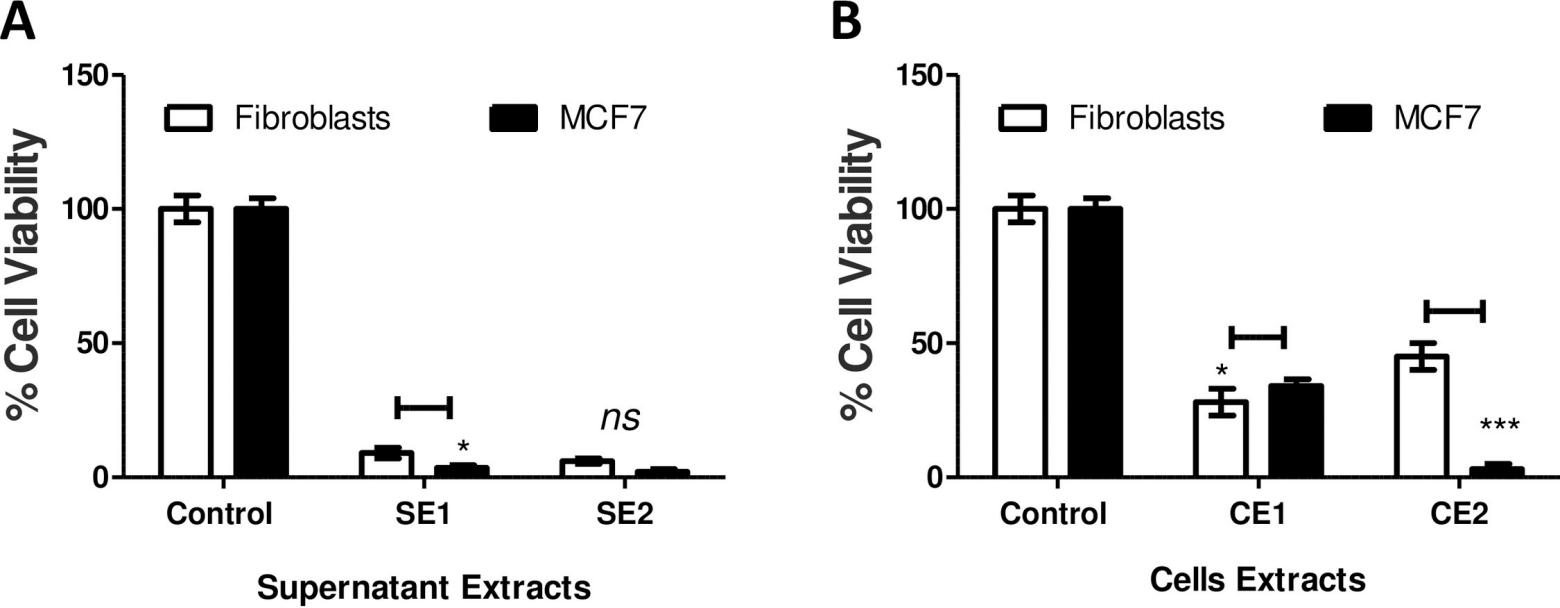
Cytotoxic activities of metabolites extracted from mammalian cell cultures. (A) Cytotoxic activities of metabolites extracted from supernatants collected from F180 (SE1) and MCF7 (SE2) cell cultures. (B) Cytotoxic activities of metabolites extracted from F180 (CE1) and MCF7 (CE2) cells separated from mammalian cell cultures. The cells were seeded in 96-well plate at a concentration of 5 × 10^4^ cells/ well in 100 μl culture medium followed by incubation with the samples overnight prior to MTT assay. The data was analyzed using two-way ANOVA and statistical significance was calculated with Bonferroni’s multiple comparisons test and significance level were indicated by asterisks (*, *P*<0.05; **, *P*<0.01: ***, *P*<0.001; ****, *P*<0.0001). The data display the mean of the percentage of the survival rate of mammalian cells ± SEM of 6 replicas.

**Fig 2:**
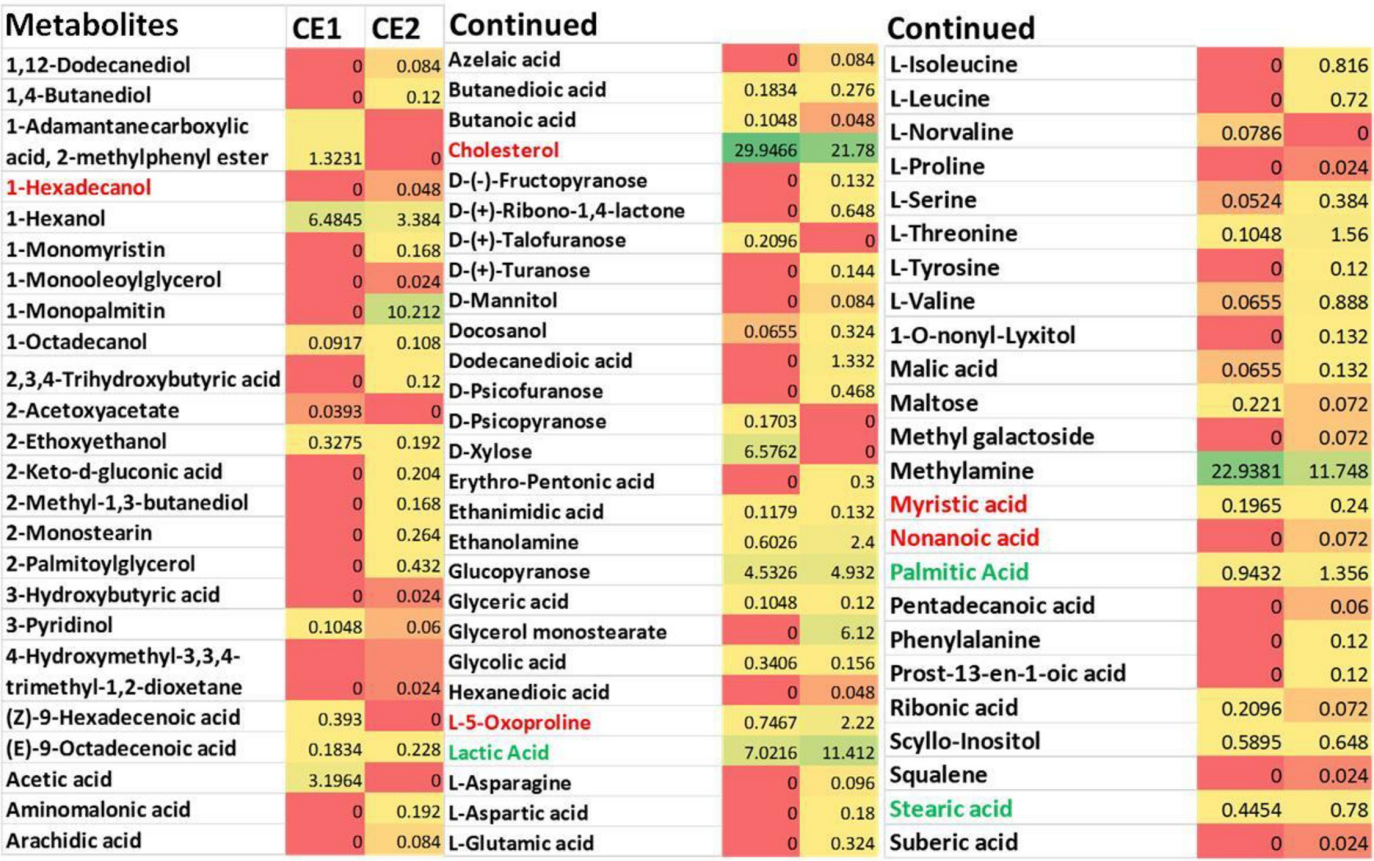
Heatmap comparing the Gas Chromatography-Mass Spectrometry (GC-MS) analysis of metabolites extracted from F180 cells (CE1) versus those extracted from MCF7 cells (CE2). The amount represented the relative percentage of a metabolite in relation to total areas of all detected metabolites in an extract. Metabolite average relative percentages of three independent replicas were displayed as colors ranging from red representing the lowest value to green for the highest value. The mammalian cells were incubated in T75 flasks until ~90% confluency, followed by centrifugation at 19000 RCF for 15 min prior to extraction by methanol with the aid of pestle.

Cultures collected from mammalian cells either MCF7 or F180 were separated by centrifugation into cells and supernatant fractions. The metabolite extracts from both supernatants (SE1 and SE2) and cells (CE1 and CE2) fractions (Table 1) were tested individually on MCF7 and F180 cells. The results indicated that all suspended extracts showed significant but variable cytotoxic activities when compared to control (Fig 1). Although extracts from both supernatants (SE1 and SE2) (Fig 1A) and fibroblasts cells (CE1) (Fig 1B) showed ~90% and 70% killing activities, respectively; this effect was non-selective as there was no significant difference in cell viability between MCF7 versus F180 cells. On the other hand, extracts from MCF7 cells (CE2) showed 95% versus 52% killing activities to MCF7 versus F180 cells, respectively (Fig 1B). The results obtained indicated that MCF7 cells metabolites extract (CE2) developed potential cytotoxic and safer activities. Metabolites extracted from MCF7 and F180 cells were analyzed by GC-MS and compared. The metabolites comparison showed the presence of many metabolites in MCF7 (CE2) but not F180 (CE1) (Fig 2). The biological activities of major metabolites identified from MCF7 but not from F180 were searched in the literature and summarized in Table 2. Most identified metabolites possess cytotoxic and oxidative catalytic activities. The results obtained indicate the importance of ligand-specific cells in targeting therapy.

**Table 2:** Reported activities of major distinctive metabolites.

Metabolite	Reported activities	Reference
**1,4-Butanediol**	It has cytotoxic activity that induces alteration in the glucose metabolism causing mitochondrial dysfunction.	[19]
**1-Monopalmitin**	It has inhibitory activities on P-glycoprotein.	[20]
**2-Keto-d-gluconic acid**	It has a role in oxidative catalytic activities.	[21]
**Azelaic acid**	It shows anti-proliferative and cytotoxic effects on human cells.	[22]
**Octacosane**	It can induce apoptosis.	[23]
**Phosphoric acid**	It shows metal stabilizer activities.	[24]
**D-(+)-ribono-1,4-lactone**	It shows cytotoxic effects.	[25]
**1-O-nonyl-lyxitol**	Toxic effects.	[26]
**Arachidic acid**	It has oxidative stability activities.	[27]
**D-(-)- fructopyranose**	It has oxidative and genotoxic effects.	[28]
**Suberic acid**	It has oxidative activity and it is associated with mitochondrial damage.	[29]
**Cyclotetradecane**	Can be involved in biofabrication of metal nanoparticles.	[30]

### Gold nanoparticles (AuNPs) developed from metabolites extracted from MCF7 cells showed promising cytotoxic activities

**Fig 3:**
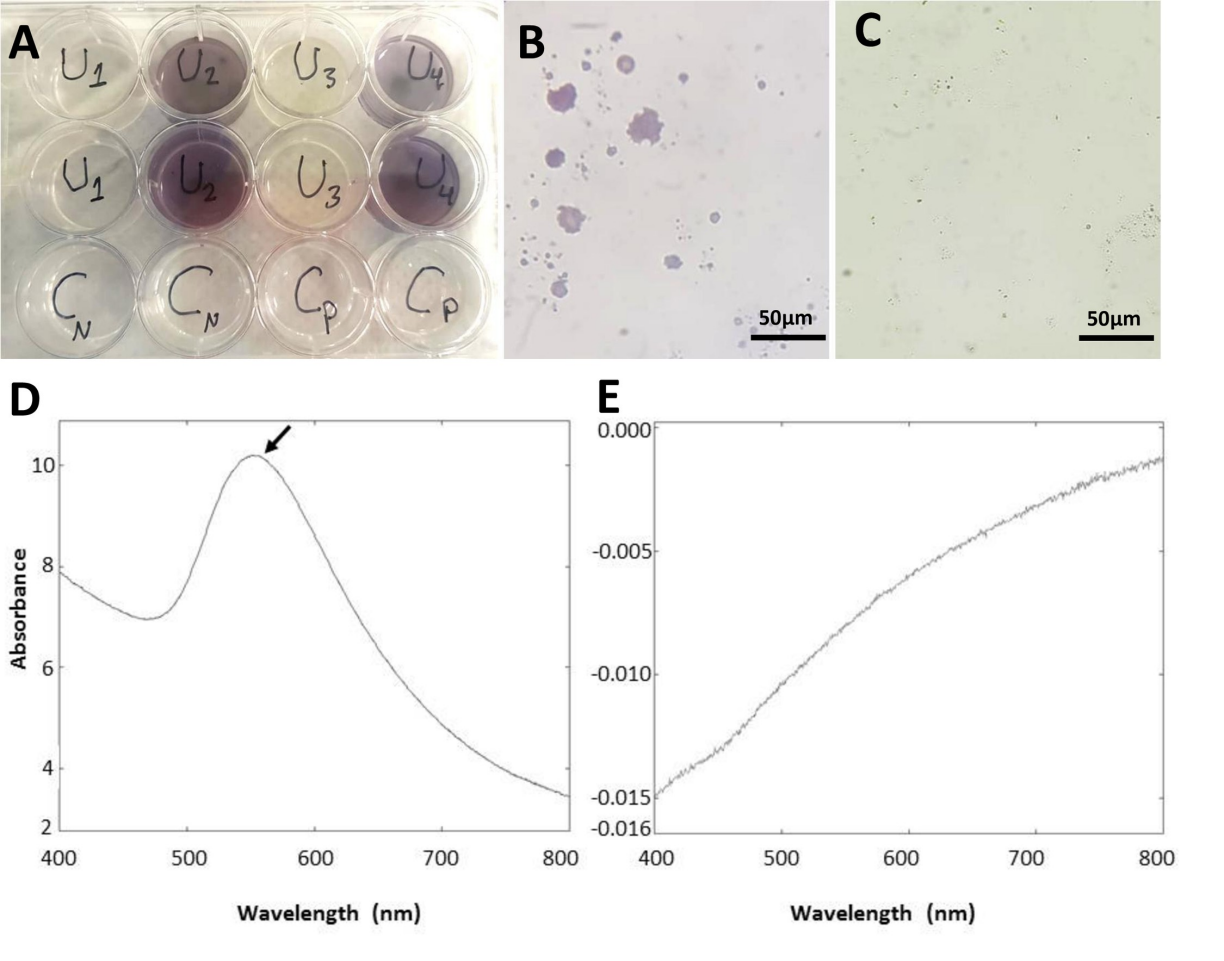
Biogenic synthesis of AuNPs and identification using UV-Vis spectroscopy. (A) Synthesis of AuNPs from mammalian cells metabolites (F180:U2 for AuNP1 and MCF7:U4 for AuNP2) versus AuNPs developed from metabolites extracted from cell-free supernatant (U1 for AuNP3 and U3 for AuNP4) and controls (Gold chloride in PBS: Cn and cells metabolites without Gold chloride: Cp). Gold chloride was incubated with metabolites extracts for 4–6 days at 37°C in 12-well plates until the development of purple color. (B and C) Light microscopy of (B) AuNPs aggregates (AuNP2) versus (C) control showed no NPs. Scale bar = 50 μm. (D and E) UV-visible spectra of (D) synthesized AuNPs (AuNP2) versus (E) control. AuNPs showed a peak at λ_max_ 550nm.

Metabolites extracted from mammalian cells and mammalian cell-free supernatants were used separately to develop AuNPs using gold chloride. The AuNPs production was indicated by the change in the color of the solution to purple (U2 and U4) [12] compared to controls (Gold chloride in PBS: Cn and cells metabolites without Gold chloride: Cp) (Fig 3A). Careful microscopy examination showed agglomerations of purple particles in wells containing metabolites-treated gold chloride (Fig 3B) while such agglomerations were absent in control groups (Fig 3C). The obtained NPs were collected by centrifugation and analyzed by UV spectroscopy at 200–900 nm range and transmission electron microscope (TEM). The synthesized AuNPs (AuNP2 and AuNP4) showed a strong peak at λ_Max_ 550 nm (Fig 3D), while no peak with the control (Fig 3E), which is consistent with previously reported data for the UV absorbance of Au^3+^ element [31].

**Fig 4:**
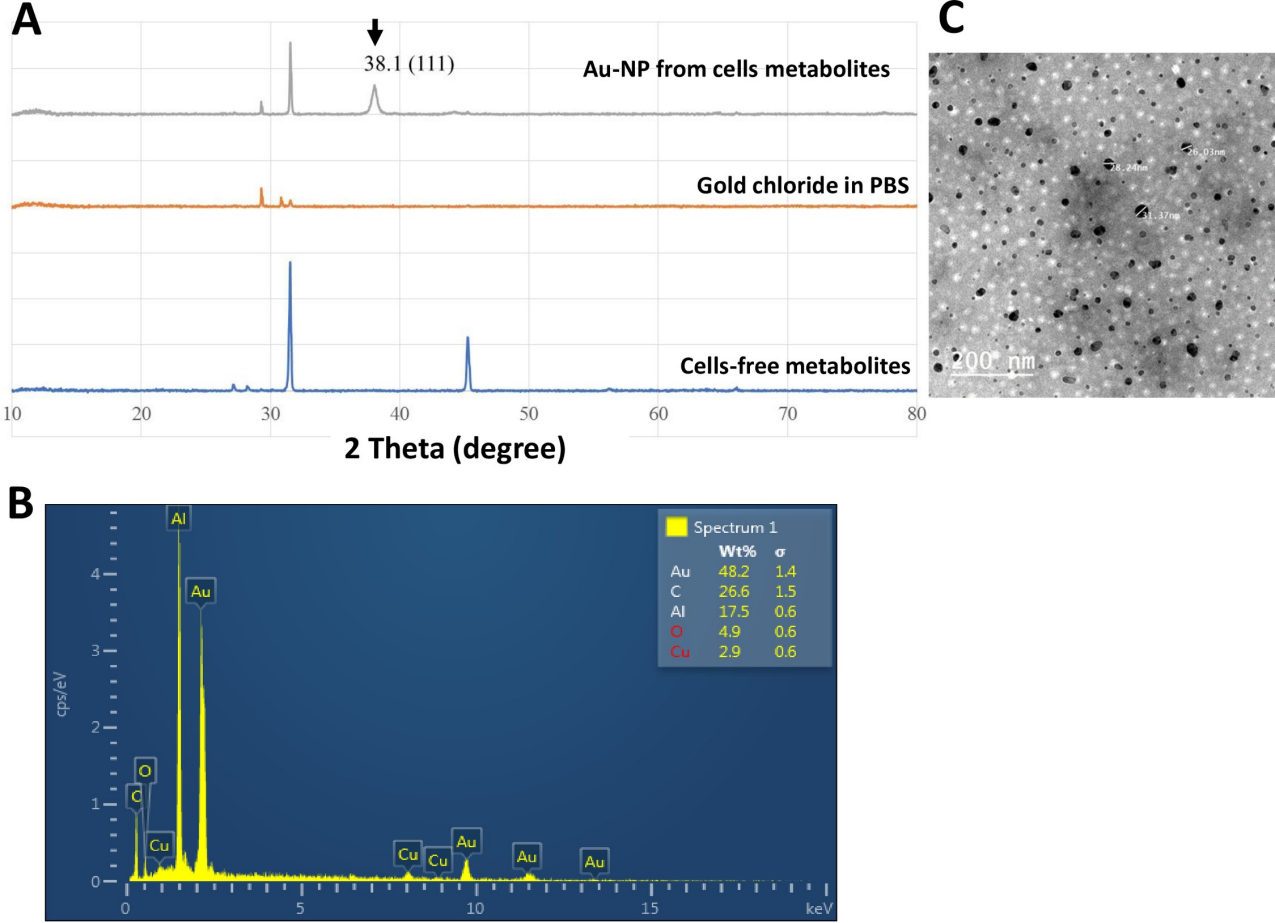
Characterization of AuNPs (AuNP2). (A) X-ray diffraction (XRD) patterns of the synthesized AuNPs. (B) SEM–EDS (energy dispersive spectroscopy) profile of the synthesized AuNPs. (C) TEM micrographs of AuNPs showed spherical-shaped particles. The samples of AuNPs were placed on standard carbon-coated copper grids (200-mesh) and air-dried for about 2h prior to measurement.

The XRD scan of the synthesized AuNPs showed a significant diffraction peak at 2θ angle: 38.1° corresponding to 111crystal plane of face-centered gold in the nanoparticles solution made from metabolites extracted from mammalian cells (AuNP2 and AuNP4), but not from metabolites solution extracted from cell-free supernatants or PBS solution, employed as control (Fig 4A). The formation of AuNPs was confirmed by the energy dispersive spectroscopy (EDS) (Fig 4B). The EDS profile showed a strong Au^3+^ signal from the NPs solution made from the metabolites extracted from mammalian cells. Furthermore, the surface morphology and topography of AuNPs analyzed by TEM showed well-defined spherical shape particles (Fig 4C). Measuring the particle size of synthesized AuNPs showed that 95% of the particles were in the size range of 28–34 nm. The average size and size distribution of the AuNPs were 30.4 nm ± 0.6 and 0.637 ± 0.156, respectively. Surface charge measurements showed that the particles carried a negative charge of 12.16 ± 1.59. The large value for size distribution can be due to partial aggregation as a result of the low surface charge and repulsive forces between the particles.

**Fig 5:**
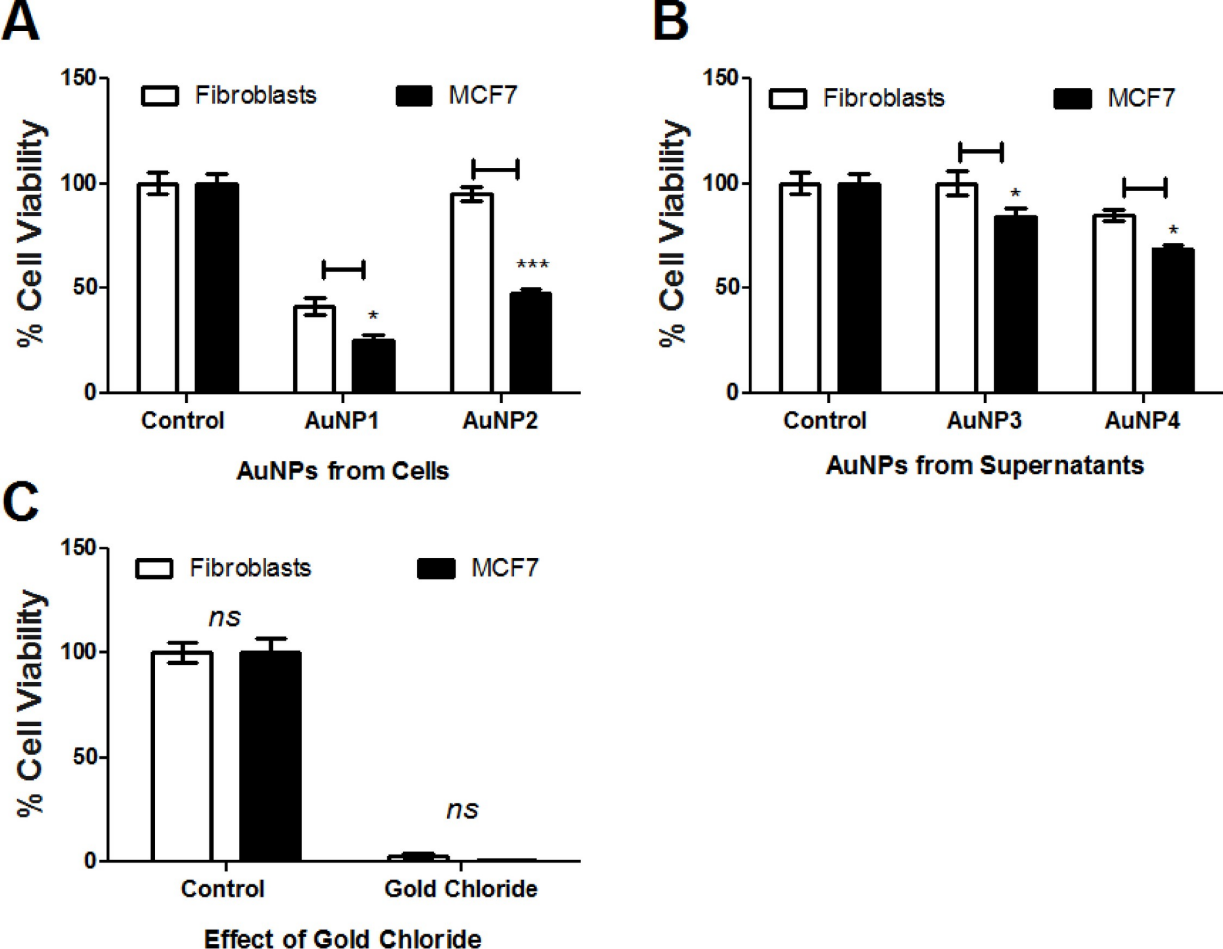
Cytotoxic activities of AuNPs developed from metabolites extracted from (A) mammalian cells (F180: AuNP1 and MCF7: AuNP2) versus (B) cells-free supernatants (AuNP3 and AuNP4) compared to (C) Gold Chloride effect. The cells were seeded in 96-well plate until confluency followed by incubation with the samples overnight prior to MTT assay. The data was analyzed using two-way ANOVA and statistical significance was calculated with Bonferroni’s multiple comparisons test and significance levels were indicated by asterisks (*, *P*<0.05; **, *P*<0.01: ***, *P*<0.001; ****, *P*<0.0001). The data display the mean of the percentage of the survival rate of mammalian cells ± SEM of 6 replicas.

The collected AuNPs were tested on fresh cancer (MCF7) and normal (fibroblasts) cells. Surprisingly, AuNPs developed from mammalian cells (AuNP1 and AuNP2) showed significant cytotoxic activities (Fig 5A), while those obtained from supernatants (AuNP3 and AuNP4) showed limited cytotoxic activities (Fig 5B). Furthermore, the AuNPs developed from MCF7 cells metabolites (AuNP2) showed significant difference in the killing effects between cancer (MCF7) versus normal (fibroblasts) cells (~52% versus 2%, *P-*value <0.001) when compared to the effects of AuNPs developed from fibroblast metabolites (AuNP1) (75% versus 65%, *P-*value <0.001) (Fig 5A). The obtained results indicate that AuNPs developed from MCF7 cell metabolites (AuNP2) showed promising anticancer activities. On the other hand, gold chloride incubated with PBS (employed as negative control) failed to develop NPs, while it caused ~100% killing effects to both mammalian cells (Fig 5C).

### Metabolomics analysis supports the prediction of responsive metabolites

#### Metabolites involved in biogenic synthesis of NPs (initiator metabolites)

**Fig 6:**
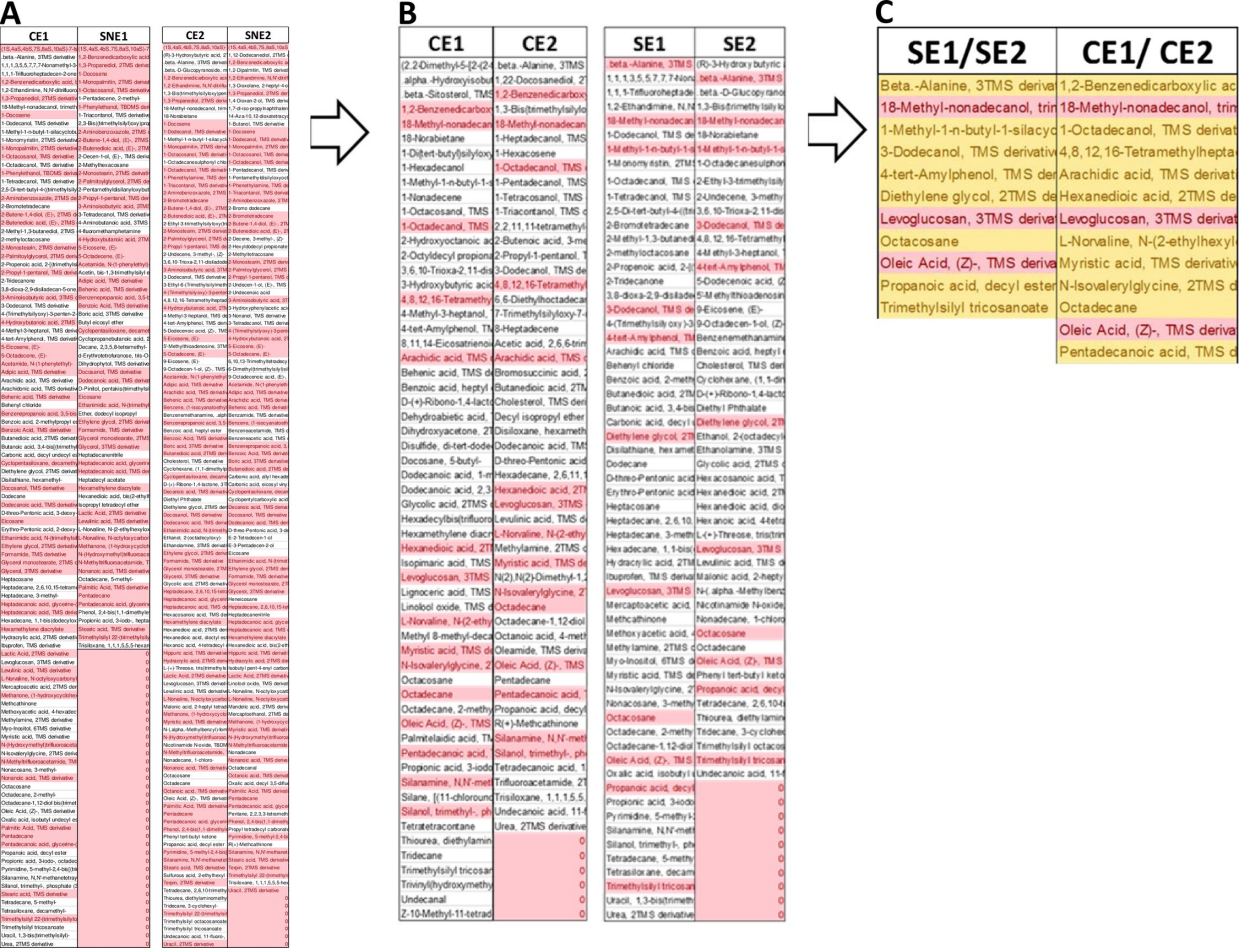
Identification of metabolites involved in biogenesis of AuNPs using GC-MS analysis. Metabolites (SNE1 and SNE2) extracted from the solution obtained after the generation of AuNPs were compared to the parent metabolites extracts (CE1 and CE2) used for their generation. The metabolite difference in the parent metabolites were selected and re-compared to metabolites extracted from cell-free supernatants (ES1 and ES3). Gold chloride was incubated with metabolites extracts dissolved in PBS for 4–6 days until the color changes. The cultures were spin down and the supernatant was collected. The supernatant was re-extracted with ethyl acetate prior to GC-MS analysis. The results obtained were compared with control extracts treated similarly but without gold chloride. (A) Metabolites comparison between gold chloride-treated versus non-treated samples. Light red-colored metabolites are shared metabolites between samples, while the uncolored metabolites are different. (B) Only metabolites identified from control samples (CE1 and CE2) but not the treated ones were compared to metabolites extracted from supernatants (SE1 and SE2). Light red-colored metabolites are shared metabolites between samples, while the uncolored metabolites are different. (C) Only shared metabolites were combined from supernatants versus cells. Light red-colored metabolites are shared metabolites between samples, while the orange metabolites are different.

To identify the metabolites involved in the formation of AuNPs, the NPs solutions obtained from F180 and MCF7 cells extracts were centrifuged and the supernatants were extracted with ethyl acetate (SNE1 and SNE2) prior to GC-MS analysis. The metabolites analyzed by GC-MS were compared to the parent metabolites extracted from both mammalian cells (employed as control, CE1 and CE2). The metabolites identified in the control but not in the AuNPs supernatants (SNE1 and SNE2) were selected and considered as NPs-initiator metabolites (reductants), since they were used up during the biogenic synthesis of NPs (Fig 6A).

The selected metabolites from CE1 and CE2 were compared to each other in order to execute the shared metabolites (Fig 6A and 6B). To ensure that the identified metabolites are only unique to the formation of AuNPs, the metabolites extracted from cell-free supernatants (SE1 and SE2) were compared to the shared ones selected from CE1 and CE2 (Fig 6B and 6C). Three metabolites were identified to be shared between all extracts (SE1 and SE2 versus CE1 and CE2) including oleic acid, levoglucosan, and 18-methyl-nonadecanol. On the other hand, the metabolites shared between CE1/ CE2 and not found in SE1/SE2 were considered as initiator and included 1,2-benzenedicarboxylic acid, bis-(2-methylpropyl) ester, 1-octadecanol, 4,8,12,16-tetramethylheptadecan-4-olide, arachidic acid, hexanedioic acid, L-norvaline, N-(2-ethylhexyloxycarbonyl)- decyl ester, myristic acid, N-isovalerylglycine, octadecane and pentadecanoic acid.

#### Metabolites involved in capping the NP

**Fig 7:**
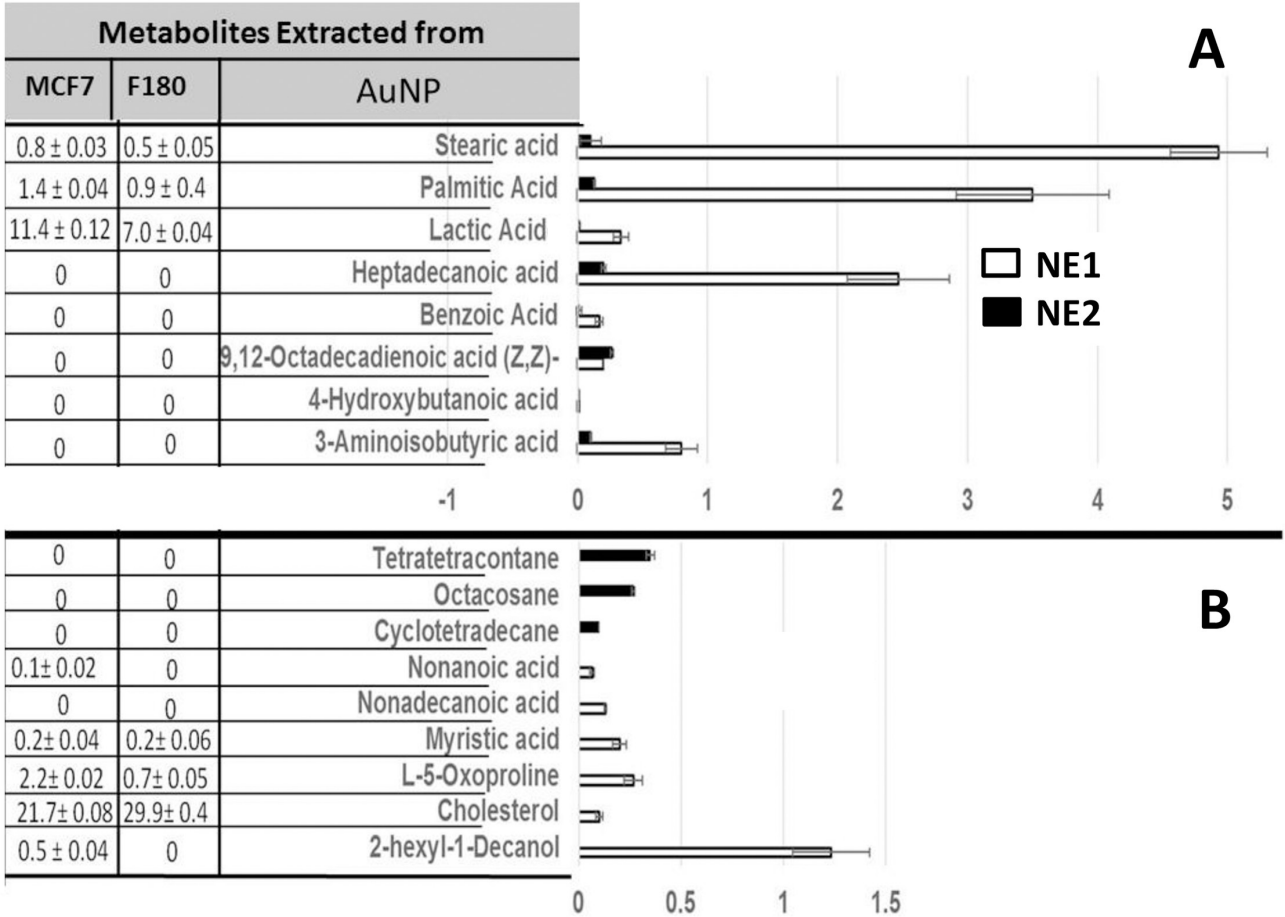
Identification of AuNPs-capping metabolites. NPs solution was centrifuged and the NPs were collected and washed by PBS two times prior to extraction using methanol and sonication for 15 min. The extracted metabolites were analyzed by GC-MS and compared to those extracted from NP-free supernatants. (A) Metabolites were identified from NPs and shared between F180 and MCF7 cell lines. (B) Unique metabolites identified from NPs developed from MCF7 versus those developed from F180. The data display the mean of the percentage of the metabolites ± SEM of three replicas. The table on the left represented the comparison between the identified metabolites from NPs to those identified in Figs 2 and 6.

To identify the capping metabolites, the nanoparticles (AuNP1 and AuNP2) obtained from both cell types were precipitated by centrifugation, washed, dried and extracted by methanol with the aid of sonication for 15 min. The extracted metabolites (NE1 and NE2) were analyzed by GC-MS and compared to each other (Fig 7), where Fig 7A indicated the metabolites shared between both NE1 and NE2, and Fig 7B indicated the different metabolites. Shared metabolites included stearic acid, palmitic acid, lactic acid, heptadecanoic acid, benzoic acid, 9,12-octadecadienoic acid (Z,Z)-, 4-hydroxybutanoic acid and 3-aminoisobutyric acid (Fig 7A). On the other hand, cyclotetradecane, octacosane, and tetratetracontane were identified only from the NE2 generated from MCF7 metabolites, while 2-hexyl-1-decanol, cholesterol, L-5-oxoproline, myristic acid, nonadecanoic acid, and nonanoic acid were identified only from those developed from fibroblasts metabolites (NE1) (Fig 7B).

#### Metabolites involved in increasing the activities of NPs (activator metabolites)

**Fig 8:**
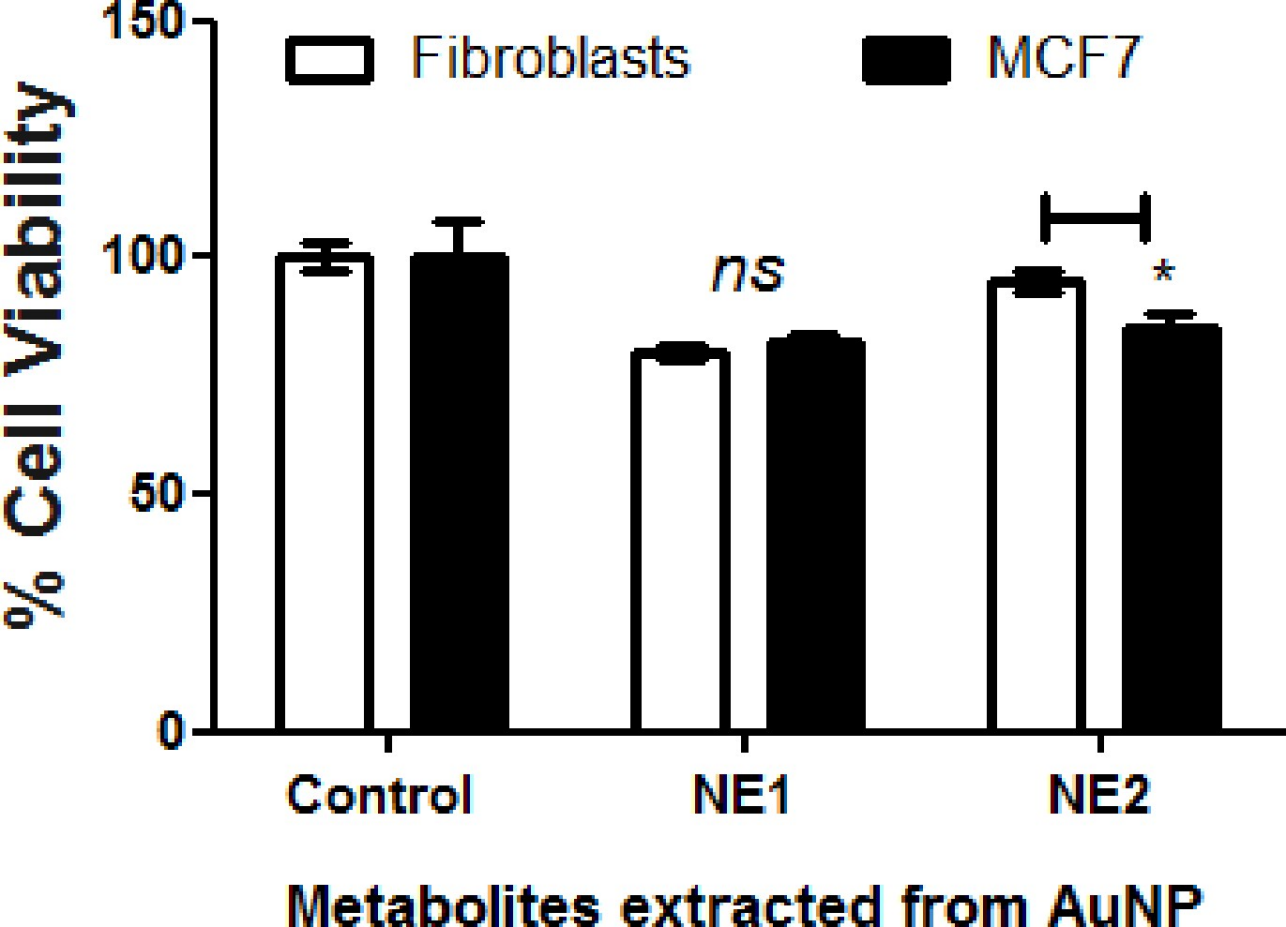
Cytotoxic activities of metabolites extracted from AuNPs. NPs solution was centrifuged and the NPs were collected and washed by PBS two times prior to extraction using methanol and sonication for 15 min. The extracted metabolites were tested on F180 and MCF7 cells using MTT assay. Mammalian cells were seeded in 96-well plate until confluency followed by incubation with the samples overnight prior to MTT assay. The data were analyzed using two-way ANOVA and statistical significance was calculated with Bonferroni’s multiple comparisons test and significance levels were indicated by asterisks (*, *P*<0.05; **, *P*<0.01: ***, *P*<0.001; ****, *P*<0.0001). The data display the mean of the percentage of the survival rate of mammalian cells ± SEM of 5 replicas.

Because the NP (AuNP2) developed from MCF7 showed promising activities when compared to those developed from F180 (AuNP1), metabolites extract identified in Fig 7 was compared to metabolites extracted from the original cells (Fig 2). Metabolites including tetratetracontane, octacosane and cyclotetradecane were identified from AuNP2 but not from the original cells or AuNP1 (Fig 7). Furthermore, the biological activities of the metabolites extracted from both AuNP1 (NE1) and AuNP2 (NE2) were compared (Fig 8). The results obtained indicated that AuNP2 metabolites (NE2) showed significant difference in the cytotoxic activities on both cell types when compared to those extracted from AuNP1 (NE1), which showed similar activities on both cells. This is consistent with the results obtained from Figs 1B and 5A; suggesting the important role of tetratetracontane, octacosane and cyclotetradecane in the variation in the activities of AuNP2 developed from MCF7. Moreover, it offers a specific approach in therapeutic target delivery [12].

## Discussion

In this study, AuNPs were synthesized using mammalian cell metabolites. The generated nanoparticles showed variable cytotoxic activities, while those generated from MCF7 showed more promising anticancer activities against MCF7. The variable activities were attributed to the variation in the metabolites profiles of both mammalian cells under study and their supernatants.

The nanoparticles generated from metabolites extracted from MCF7 showed significant differences in the cytotoxic activities between both cancer and normal cells, while those generated from F180 or cells-free supernatants did not. It has been reported that AuNPs demonstrated different cytotoxic effects on different mammalian cells [32]. Although the variable cytotoxic activities on different mammalian cells have been attributed to the size and level of aggregations [8], our results showed that the origin of reductants is another factor. The metabolites extracted from MCF7 cells caused significant anticancer activities on MCF7 when compared to F180, while those extracted from F180 cells showed no differences in cytotoxic activities on both cell types. Similarly, AuNPs generated from MCF7 caused variable activities on both cell types, while those generated from F180 are not. We attributed this difference in activities to the presence of variable metabolites species or levels. Many different bio-molecular chemicals were reported to be involved in AuNPs formation and stabilization into smaller nanoclusters. These chemicals include amino acids, protein side chains, glutathione, phospholipids, and many more [6].

Metabolites that may cap or conjugate to NPs were extracted and showed that some metabolites identified only from AuNPs generated from MCF7 including tatratetracontane, octacosane and cyclotetradecane, while other metabolites were related to F180 including hexyldecanol, oxoproline, myristic acid and nonanoic acid. Saturated hydrocarbons such as octacosane have been reported to induce apoptosis [23]. On the other hand, fatty acids level were 10–20 times higher in case of NPs generated from F180 when compared to those identified from AuNPs generated from MCF7. The presence of a high level of fatty acids may be responsible for the higher non-selective cytotoxic activities of AuNPs generated from F180. Consistently, the cytotoxicity due to free fatty acids was reported [33]. Although few metabolites with cytotoxic activities were identified from MCF7 but not from F180, none of them was detected from AuNPs themselves. These metabolites included azelaic acid [34], D-(+)-ribono-1,4-lactone [25], 1-O-nonyl-lyxitol [26] and phosphoric acid [35].

The metabolites predicted from mammalian cells and involved in biogenic synthesis of AuNPs were mainly alcohols and acids. These metabolites included 1,2-benzenedicarboxylic acid, bis(2-methylpropyl) ester, octadecanol, arachidic acid, hexanedioic acid, myristic acid and pentadecanoic acid. For example, 1,2-benzenedicarboxylic acid, bis(2-methylpropyl) ester is a reducing agent [36]. On the other hand, oleic acid has been identified in all metabolites extracts. Oleic acid can modify the electronic structure of AuNPs [37], stabilize AuNPs [38] and affect the cytotoxic activities of AuNPs [39] but cannot initiate the synthesis of NPs.

The use of plant metabolites [40] or bacterial metabolites [41] to induce the reduction of gold to nanoparticles has been reported before. However, the use of mammalian cell metabolites to generate gold nanoparticles is the first. The results obtained in this study may help to design a well-controlled delivery system after optimization of size and aggregation level using fractional size exclusion chromatography. Furthermore, optimization of the metabolites capping the nanoparticles will be of important value to identify the metabolites with significant impact on the developed nanoparticles. Although the focus of this manuscript was the breast cancer cells (MCF7), we believe that further future investigations are required. Testing the biological activities of NPs developed from metabolites extracted from different breast cancer fractions such as the cell membrane and cytosol will help to narrow down the metabolites responsible for the variable activities. Repeating the NPs synthesis using different cancer cells will help to either generalize or specify our final conclusion. Moreover, fractionation of the extracted metabolites used to develop the nanoparticles by column chromatography or HPLC followed by NPs synthesis and activities will also help to identify the major responsive metabolites.

## Conclusions

In conclusion, it has been demonstrated that metabolites extracted from mammalian cells are capable of producing AuNPs extracellularly and the generated nanoparticles are quite stable. The developed nanoparticles showed variable cytotoxic activities depend on the used cell types. Nanoparticles generated from MCF7 metabolites showed promising anticancer activities on their own. This variation in anticancer activities was attributed to the differences in the metabolites and/ or their levels. This report will also lead to the development of a rational biosynthetic procedure from other mammalian cells or their compartments for several and unique biomedical applications.

## Abbreviations

AuNP: gold nanoparticle
CE: cell extract
F180: normal fibroblast cells
MCF7: breast cancer cells
NE: nanoparticle extract
NP: nanoparticle
SE: supernatant extract
SEM: scanning electron microscopy
SNE: supernatant collected from NP
TEM: transmission electron microscopy

## References

[1] Dykman L, Khlebtsov N. Gold nanoparticles in biomedical applications: Recent advances and perspectives. Chem Soc Rev. 2012;41(6):2256–82. 10.1039/c1cs15166e 22130549

[2] Freitas de Freitas L, Varca GHC, Dos Santos Batista JG, Benévolo Lugão A. An overview of the synthesis of gold nanoparticles using radiation technologies. Nanomaterials (Basel). 2018;8(11):939 10.3390/nano8110939 30445694 PMC6266156

[3] He S, Guo Z, Zhang Y, Zhang S, Wang J, Gu N. Biosynthesis of gold nanoparticles using the bacteria *Rhodopseudomonas capsulata*. Mater Lett. 2007;61(18):3984–7. 10.1016/j.matlet.2007.01.018.

[4] Lengke MF, Fleet ME, Southam G. Morphology of gold nanoparticles synthesized by filamentous cyanobacteria from gold(i)−thiosulfate and gold(iii)−chloride complexes. Langmuir. 2006;22(6):2780–7. 10.1021/la052652c 16519482

[5] Husseiny MI, El-Aziz MA, Badr Y, Mahmoud MA. Biosynthesis of gold nanoparticles using *Pseudomonas aeruginosa*. Spectrochim Acta A. 2007;67(3):1003–6. 10.1016/j.saa.2006.09.028. 17084659

[6] Drescher D, Traub H, Büchner T, Jakubowski N, Kneipp J. Properties of in situ generated gold nanoparticles in the cellular context. Nanoscale. 2017;9(32):11647–56. 10.1039/c7nr04620k 28770918

[7] Cui W, Li J, Zhang Y, Rong H, Lu W, Jiang L. Effects of aggregation and the surface properties of gold nanoparticles on cytotoxicity and cell growth. Nanomedicine. 2012;8(1):46–53. 10.1016/j.nano.2011.05.005 21658475

[8] Coradeghini R, Gioria S, García CP, Nativo P, Franchini F, Gilliland D, et al Size-dependent toxicity and cell interaction mechanisms of gold nanoparticles on mouse fibroblasts. ‎ Toxicol Lett. 2013;217(3):205–16. 10.1016/j.toxlet.2012.11.022 23246733

[9] Yoo J, Park C, Yi G, Lee D, Koo H. Active targeting strategies using biological ligands for nanoparticle drug delivery systems. Cancers (Basel). 2019;11(5):640 10.3390/cancers11050640 . 31072061 PMC6562917

[10] Wu J-J, Omar HA, Lee Y-R, Teng Y-N, Chen P-S, Chen Y-C, et al 6-Shogaol induces cell cycle arrest and apoptosis in human hepatoma cells through pleiotropic mechanisms. European Journal of Pharmacology. 2015;762:449–58. 10.1016/j.ejphar.2015.06.032 26101062

[11] Soliman S, Li XZ, Shao S, Behar M, Svircev AM, Tsao R, et al Potential mycotoxin contamination risks of apple products associated with fungal flora of apple core. Food Control. 2015;47:585–91. 10.1016/j.foodcont.2014.07.060.

[12] Yeh Y-C, Creran B, Rotello VM. Gold nanoparticles: preparation, properties, and applications in bionanotechnology. Nanoscale. 2012;4(6):1871–80. Epub 2011/11/10. 10.1039/c1nr11188d . 22076024 PMC4101904

[13] Al-Nuairi AG, Mosa KA, Mohammad MG, El-Keblawy A, Soliman S, Alawadhi H. Biosynthesis, characterization, and evaluation of the cytotoxic effects of biologically synthesized silver nanoparticles from *Cyperus conglomeratus* root extracts on breast cancer cell line MCF-7. Biol Trace Elem Res. 2019:1–10. 10.1007/s12011-019-01791-7 31267442

[14] Hamdy R, Fayed B, Hamoda AM, Rawas-Qalaji M, Haider M, Soliman SSM. Essential Oil-Based Design and Development of Novel Anti-Candida Azoles Formulation. Molecules (Basel, Switzerland). 2020;25(6):1463 10.3390/molecules25061463 . 32213931 PMC7146627

[15] Soliman S, Hamoda AM, El-Shorbagi A-NA, El-Keblawy AA. Novel betulin derivative is responsible for the anticancer folk use of *Ziziphus spina-christi* from the hot environmental habitat of UAE. J Ethnopharmacol. 2019;231:403–8. 10.1016/j.jep.2018.11.040 30508621

[16] Sun H, Jia J, Jiang C, Zhai S. Gold Nanoparticle-Induced Cell Death and Potential Applications in Nanomedicine. Int J Mol Sci. 2018;19(3):754 10.3390/ijms19030754 . 29518914 PMC5877615

[17] Khatibi S, Taban ZF, Roushandeh AM. In vitro evaluation of cytotoxic and antiproliferative effects of *Portulaca oleracea* ethanolic extracton on hela cell line. Gene Cell Tissue. 2016;4(1):e13301.

[18] Semreen MH, Soliman SSM, Saeed BQ, Alqarihi A, Uppuluri P, Ibrahim AS. Metabolic profiling of candida auris, a newly-emerging multi-drug resistant candida species, by GC-MS. Molecules. 2019;24(3):399 10.3390/molecules24030399 30678308 PMC6384714

[19] Nocca G, Martorana GE, De Sole P, De Palma F, Callà C, Corsale P, et al Effects of 1, 4‐butanediol dimethacrylate and urethane dimethacrylate on HL‐60 cell metabolism. Eur J Oral Sci. 2009;117(2):175–81. 10.1111/j.1600-0722.2008.00606.x 19320727

[20] Konishi T, Satsu H, Hatsugai Y, Aizawa K, Inakuma T, Nagata S, et al Inhibitory effect of a bitter melon extract on the P-glycoprotein activity in intestinal Caco-2 cells. Br J Pharmacol. 2004;143(3):379–87. Epub 2004/09/06. 10.1038/sj.bjp.0705804 . 15351776 PMC1575343

[21] Zeng W, Cai W, Liu L, Du G, Chen J, Zhou J. Efficient biosynthesis of 2-keto-D-gluconic acid by fed-batch culture of metabolically engineered *Gluconobacter japonicus*. Synth Syst Biotechnol. 2019;4(3):134–41. 10.1016/j.synbio.2019.07.001 31384676 PMC6661466

[22] Fitton A, Goa KL. Azelaic acid. Drugs. 1991;41(5):780–98. 10.2165/00003495-199141050-00007 1712709

[23] Figueiredo CR, Matsuo AL, Pereira FV, Rabaça AN, Farias CF, Girola N, et al Pyrostegia venusta heptane extract containing saturated aliphatic hydrocarbons induces apoptosis on B16F10-Nex2 melanoma cells and displays antitumor activity in vivo. Pharmacogn Mag. 2014;10(Suppl 2):S363–S76. 10.4103/0973-1296.133284 . 24991116 PMC4078348

[24] Kim N-H, Lim J-H, Kim S-Y, Chang E-G. Effects of phosphoric acid stabilizer on copper and tantalum nitride CMP. Mater Lett. 2003;57(29):4601–4. 10.1016/S0167-577X(03)00368-9.

[25] Kitajima J, Ishikawa T, Tanaka T, Ida Y. Water-soluble constitutents of fennel. IX. glucides and nucleosides. Chem Pharm Bull. 1999;47(7):988–92. 10.1248/cpb.47.988

[26] Gan W, Zhou M, Xiang Z, Han X, Li D. Combined effects of nonylphenol and bisphenol a on the human prostate epithelial cell line RWPE-1. Int J Environ Res Public Health. 2015;12(4):4141–55. 10.3390/ijerph120404141 . 25874684 PMC4410238

[27] Araseki M, Yamamoto K, Miyashita K. Oxidative stability of polyunsaturated fatty acid in phosphatidylcholine liposomes. Biosci Biotechnol Biochem. 2002;66(12):2573–7. 10.1271/bbb.66.2573 12596850

[28] Czubatka-Bienkowska A, Macieja A, Sarnik J, Witczak ZJ, Poplawski T. The oxidative induction of DNA lesions in cancer cells by 5-thio-d-glucose and 6-thio-d-fructopyranose and their genotoxic effects. Part 3. Bioorg Med Chem Lett. 2017;27(5):1210–4. 10.1016/j.bmcl.2017.01.011 28094181

[29] Largilliere C, Vianey-Saban C, Fontaine M, Bertrand C, Kacet N, Farriaux J-P. Mitochondrial very long chain acyl-CoA dehydrogenase deficiency-a new disorder of fatty acid oxidation. Arch Dis Child. 1995;73(2):F103–F5. 10.1136/fn.73.2.f103 PMC2528513 7583594

[30] Jayaseelan C, Gandhi PR, Rajasree SRR, Suman TY, Mary RR. Toxicity studies of nanofabricated palladium against filariasis and malaria vectors. Environ Sci Pollut Res. 2018;25(1):324–32. 10.1007/s11356-017-0428-x 29034429

[31] Shang Y, Min C, Hu J, Wang T, Liu H, Hu Y. Synthesis of gold nanoparticles by reduction of HAuCl4 under UV irradiation. Solid State Sci. 2013;15:17–23. 10.1016/j.solidstatesciences.2012.09.002.

[32] Chueh PJ, Liang R-Y, Lee Y-H, Zeng Z-M, Chuang S-M. Differential cytotoxic effects of gold nanoparticles in different mammalian cell lines. J Hazard Mater. 2014;264:303–12. 10.1016/j.jhazmat.2013.11.031 24316248

[33] Siegel I, Liu TL, Yaghoubzadeh E, Keskey TS, Gleicher N. Cytotoxic effects of free fatty acids on ascites tumor cells2. JNCI. 1987;78(2):271–7. 10.1093/jnci/78.2.271 3468290

[34] N Z, R V, L S, O S, R S. Cytotoxic activity of azelaic acid against human melanoma primary cultures and established cell lines. Anticancer Res. 1990;10(6):1599–602. 2285231

[35] Prado M, Silva EJNLd, Duque TM, Zaia AA, Ferraz CCR, Almeida JFAd, et al Antimicrobial and cytotoxic effects of phosphoric acid solution compared to other root canal irrigants. J Appl Oral Sci. 2015;23:158–63. 10.1590/1678-775720130691 26018307 PMC4428460

[36] Ghosh S, Derle A, Ahire M, More P, Jagtap S, Phadatare SD, et al Phytochemical analysis and free radical scavenging activity of medicinal plants *Gnidia glauca* and *Dioscorea bulbifera*. PLoS One. 2013;8(12):e82529–e. 10.1371/journal.pone.0082529 . 24367520 PMC3867356

[37] de la Presa P, Multigner M, de la Venta J, García MA, Ruiz-González ML. Structural and magnetic characterization of oleic acid and oleylamine-capped gold nanoparticles. J Appl Phys. 2006;100(12):123915 10.1063/1.2401314

[38] Sadrolhosseini AR, Abdul Rashid S, Zakaria A. Synthesis of gold nanoparticles dispersed in palm oil using laser ablation technique. J Nanomater. 2017;2017:5 10.1155/2017/6496390

[39] Poursharifi M, Wlodarczyk MT, Mieszawska AJ. Nano-based systems and biomacromolecules as carriers for metallodrugs in anticancer therapy. Inorganics. 2018;7(1):2 10.3390/inorganics7010002

[40] Kasthuri J, Veerapandian S, Rajendiran N. Biological synthesis of silver and gold nanoparticles using apiin as reducing agent. Colloids Surf B Biointerfaces. 2009;68(1):55–60. 10.1016/j.colsurfb.2008.09.021 18977643

[41] Rajeshkumar S. Anticancer activity of eco-friendly gold nanoparticles against lung and liver cancer cells. J Genet Eng Biotech. 2016;14(1):195–202. 10.1016/j.jgeb.2016.05.007. PMC6299896 30647615

