# Correction: Effective targeting of breast cancer cells (MCF7) via novel biogenic synthesis of gold nanoparticles using cancer-derived metabolites

**DOI:** 10.1371/journal.pone.0350791

**Published:** 2026-06-04

**Authors:** Sameh S. M. Soliman, Tasneem B. Alhamidi, Shifaa Abdin, Ahmed M. Almehdi, Mohammad H. Semreen, Razan B. Alhumaidi, Sarra B. Shakartalla, Mohamed Haider, Mohamed I. Husseiny, Hany A. Omar

Contrary to the Data Availability statement in [1], the individual-level underlying data supporting the article’s results were not provided. The corresponding author stated that the raw data underlying the individual replicate datasets for Fig 2 are no longer available, but provided averaged data supporting Fig 2 and the underlying data supporting Figs 1 and 3-8.

With this Correction, all relevant data, with the exception of the individual-level data underlying Fig 2, are now provided in S1 File.

The Data Availability statement in [1] is updated to: All relevant data are within the paper and its Supporting Information files.

## Supporting information

S1 File Raw data underlying Figs 1 and 3–8; averaged data underlying Fig 2.
(ZIP)
